# Genome Sequences of a Green-Colored Chlorobium phaeovibrioides Strain Containing Two Plasmids and a Closely Related Plasmid-Free Brown-Colored Strain

**DOI:** 10.1128/MRA.01172-19

**Published:** 2020-01-09

**Authors:** Daria I. Boldyreva, Vladislav V. Babenko, Alexandra V. Kanygina, Olga N. Lunina, Maria A. Letarova, Elena S. Kostryukova, Alexander S. Savvichev, Vladimir M. Gorlenko, Andrey V. Letarov

**Affiliations:** a Federal Medical Biological Agency, Federal Research and Clinical Centre of Physical-Chemical Medicine, Moscow, Russia; b Research Centre of Biotechnology, Russian Academy of Sciences, Moscow, Russia; Portland State University

## Abstract

Here, we report the draft genome sequences of the green sulfur bacterium Chlorobium phaeovibrioides strains GrTcv12 and PhvTcv-s14, isolated from the chemocline zone from meromictic Lake Trekhtzvetnoe, separated from the White Sea, in Russia. This is the first report showing the presence of plasmids containing antiphage systems in the *Chlorobium* sp. genome.

## ANNOUNCEMENT

In Lake Trekhtzvetnoe (Russia), brown-colored Chlorobium phaeovibrioides PhvTcv-s14 is located in the upper layers of the chemocline zone above green-colored Chlorobium phaeovibrioides GrTcv12, which has particular phenotype distinctions (1). Both strains were isolated from the chemocline of Lake Trekhtzvetnoe, which is the smallest meromictic lake with constant stratification. This lake has constant hydrological and biological stratification over a short water column and a high density of *C. phaeovibrioides* in the chemocline layer (2).

The strains were grown as described previously (1). The DNA was extracted by using the Wizard DNA extraction kit (Promega Corporation, USA) and size selected with optimized solid‐phase reversible immobilization (SPRI) beads (3). The long reads were generated with MinION sequencing (Oxford Nanopore Technologies, UK). The sequencing libraries were prepared using the ligation sequencing kit (catalog number SQK-LSK109) and native barcoding expansion kit (catalog number EXP-NBD114) and run in a FLO-MIN106 flow cell. Reads were base called using Albacore v1.2.5 and trimmed and demultiplexed with Porechop v0.2.1 using default parameters (4). The short-read whole-genome sequencing (WGS) for each strain was generated using the Ion Torrent PGM (Life Technologies, USA) sequencing platform with the Ion Xpress plus fragment library kit (Life Technologies) using 400-bp chemistry. Prinseq lite v0.20.4 was used for read-quality (Q > 20) trimming. *De novo* assembly was performed by hybrid assembler Unicycler (v0.4.8) (5) using default parameters. Identification of the protein-coding sequences and primary annotation were performed using PGAP v4.7 (6). All relevant sequencing and assembly statistics are summarized in Table 1.

**TABLE 1:** Sequencing and assembly statistics for *C. phaeovibrioides* PhvTcv-s14 and GrTcv12

Parameter	Data for strain:					
	PhvTcv-s14	GrTcv12				
		pl1	pl2	Linear contig	Circular chromosome	Whole genome
Ion Torrent PGM						
No. of generated reads	404,071					1,001,673
Mean read length (bp)	218					216
MinION						
No. of generated reads	199,320					348,897
Mean read length (bp)	7,351					5,090
Sequence length (bp)	2,017,051	90,349	35,536	132,296	2,055,276	
G+C content (%)	53	49.4	50.2	52.6	52.7	
No. of CDSs^*a*^	1,842	77	29	139	1,704	
Coverage (×)	750					800
No. of genes	1,903					2,207
No. of pseudogenes	254					202
No. of RNA genes	61					56

a CDSs, coding DNA sequences.

PhvTcv-s14 was assembled as a single circular chromosome. GrTcv12 was assembled as two chromosome contigs (circular and linear) and two plasmids (pl1 and pl2). The coverages of the chromosome, pl1, and linear contig were almost identical, while the coverage of pl2 exceeded the chromosome coverage by a factor of 4.5. The pl2 plasmid was previously detected in a shotgun metagenome of the Lake Trekhtzvetnoe chemocline water due to its elevated coverage (2).

Among known bacterial genomes of the phylum *Chlorobi*, only Prosthecochloris aestuarii has been identified to carry a plasmid (NCBI reference sequence accession number NC_011061). Thus, we can suppose that the presence of plasmids in the genome is not typical for *Chlorobium* spp. Plasmid pl1 contains genes related to the type I-F CRISPR-Cas system and serine/threonine proteins (7, 8). Plasmid pl2 contains genes related to AbiEii/AbiGii toxin family proteins and to the restriction-modification system. The linear contig also encodes a bacteriophage exclusion system, BREX, a prophage-related region, and some housekeeping genes. The presence of multiple (at least 5) antiphage systems in the *C. phaeovibrioides* GrTcv12 genome suggests a significant load of the phage infection in the recent evolutionary history of this strain, which is in good agreement with the previously obtained data (9).

**FIG 1:**
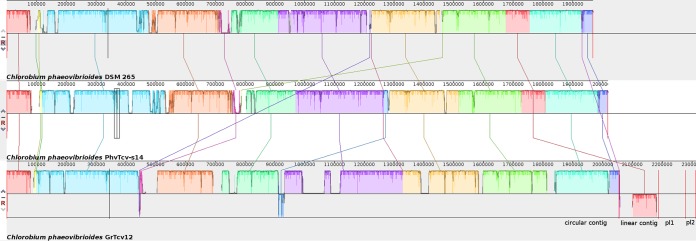
Mauve genome comparison between the reference genome *C. phaeovibrioides* DSM 265 (upper) and the studied genomes *C. phaeovibrioides* PhvTcv-s14 (middle) and *C. phaeovibrioides* GrTcv12 (lower). Each contiguously colored region is a locally colinear block (LCB), a region without rearrangement of homologous backbone sequence. LCBs identified by Mauve are color coded; links between LCBs are indicated by the thin colored lines. LCBs below a genome’s center line are in the reverse complement orientation relative to the reference DNA sequence. Unmatched regions within an LCB indicate the presence of a strain-specific sequence. The contigs are separated by red lines. The scale is in nucleotides.

Comparative analysis of GrTcv12 and PhvTcv-s14 with *C. phaeovibrioides* DSM 265 (10) using Mauve (11) shows that these genomes are very close to each other (Fig. 1).

The average nucleotide identity (ANI) and digital DNA-DNA hybridization (dDDH) values were calculated using the ANI calculator (12) and the Genome-to-Genome Distance Calculator (GGDC) v 2.1 (13), respectively. In comparison with *C. phaeovibrioides* DSM 265, the ANI values of strains GrTcv12 and PhvTcv-s14 were 98.99%, and 98.93% and the dDDH values were 91.10% and 89.50%, respectively. The calculated values exceeded the proposed boundary values for species delineation (ANI, 95% to 96%; dDDH, 70%) (14), which suggests that strains GrTcv12 and PhvTcv-s14 are novel strains of the known species Chlorobium phaeovibrioides. Also, they have similarities with C*. phaeovibrioides* GrKhr17 and BrKhr17 from the neighboring Lake Bolshye Khruslomeny (15). Further study of green sulfur bacteria (GSB) in this area can give insight into how phages influence bacterial genome changes during evolution.

The genomes of GrTcv12 and PhvTcv-s14 contain the *gvp* genes that are required for gas vesicle formation. Also, both genomes contain genes required for biosynthesis of Bchl *a*, *b*, *c*, and *d* and genes which are responsible for the biosynthesis of isorenieratene and chlorobactin (16, 17). There are *bciD* genes required for Bchl *e* synthesis in the PhvTcv-s14 genome. Sequencing and analysis of these bacteria revealed genomic determinants of the particular phenotypes of new strains of Chlorobium phaeovibrioides from Arctic meromictic lakes.

### Data availability.

Genome sequence data of Chlorobium phaeovibrioides PhvTcv-s14 and Chlorobium phaeovibrioides GrTcv12 were deposited into NCBI GenBank under BioSample accession numbers SAMN09466660 and SAMN09466659, respectively, and under BioProject accession number PRJNA438928. Raw sequence reads are available under the SRA accession numbers SRR10277008 (MinION) and SRR10277009 (IonTorrent PGM) for Chlorobium phaeovibrioides PhvTcv-s14 and SRR10277006 (MinION) and SRR10277007 (IonTorrent PGM) for Chlorobium phaeovibrioides GrTcv12.
